# Precautions during Anæsthesia

**Published:** 1875-03

**Authors:** 


### ARTICLE VI.

### *Precautions During Anæsthesia.*

The following extracts are from a note appended to a letter addressed to the *British Medical Journal*, by Dr. Bigelow, of Boston:

Under these circumstances, a few practical suggestions, in a familiar form, however superfluous or even trite to a part of the surgical world, may perhaps not inappropriately serve as a record of the current views and practice of etherization in the hospital with which I am connected—which has, perhaps, a larger experience than any other of this form of anæsthesia.

1. Accept the odor and bulk of ether as a cheap compromise for the safety of the patient and the confidence it gives the operator.

2. Believe that the anæsthetic effects, whether pleasant or objectionable, do not differ materially from those of chloroform.

3. Recognize the fact that, while chloroform may kil without warning, ether never does.

4. Aim at anæsthesia by inebriation, not by asphyxia With ether vapor, insure air to the patient. Though he struggle at the beginning, if he is not rigid, or too livid, it is safe to compel inhalation; but if you can devote more time to the process, the resistance will be often less.

(Before etherizing, remove false teeth, and loosen a tight dress.)

5. Use, and let hospital attendants use, a good sized bell shaped sponge; and then it may be a question of less rather than more air. The various forms of apparatus which restrict or graduate the quantity of air require more attention and more assistance. Of these, a close bag is the worst. If the sponge is damp, it retains ether better, while the vapor is perhaps a little softer than when absolutely pure. The ready ignition of the latter suggests the precaution of moistening with water the skin and saturated linen before employing near the face even galvano cautery. (The gravitation of the vapor makes it practically safe by night, if lamps are held above it.)

Keep the pulse in hand; at any rate, examine it often. When the pulse is right the patient is so. With chloroform the pulse may be right and the patient wrong. If slow or feeble, or if the patient snores more than he need, save his strength by giving air—at any rate, until the pulse comes up; but renew the ether before he is sensible of pain. If the pulse shows that he is suddenly faint, lay him down and give him air. Faintness not unfrequently results from nausea, and is relieved by vomiting. In a case of doubtful pulse, a contractile pupil re-assures the operator; a dilated pupil renders him more cautious.

7. If the patient is livid and rigid, give him air.

8. If his glottis contracts, give him air.

9. If he breathes badly, put the finger inside the cheek, to admit air over the base of the tongue.

10. Should he vomit, of which there is usually timely notice, give the matter free exit by turning the patient, if recumbent, well to one side. Although there is less nausea with an empty stomach, it is not well to starve a patient about to encounter a protracted operation.

11. From time to time evacuate the tracheal mucus from the fauces, during an expiration, with a sponge held in dressing forceps.

12. In operations about the nose and mouth, give, for convenience, a powerful dose before beginning. Impregnate the whole circulation to the degree it usually attains in the middle of a long operation. The patient is then easily kept quiet. Otherwise, a volume of fresh blood may find its way to the brain, and suddenly revive him. Let the repeated dose, also be heavy.

13. In these operations, expect blood in the trachea, and evacuate it like the mucus—but, by reason of its quantity, more promptly,

14. Indeed, if such an operation promises much blood, have a tracheotomy tube ready, with hooks to hold the incision open while they compress the veins, so that the tube can be entered by a cut or two in a few seconds.

15. Or insert the tube before operation, and put a sponge in the pharynx. The patient may then be etherized through the tube. I have had occasion to resort to these expedients.

16. In artificial respiration, act with the patient, and not against him. He will not cease to breathe at once, and wholly. Enjoin silence; watch the first attempt at inspiration, and at the expiration compress the thorax, aiding in elastic reaction, if absolutely necessary, by Silvester's or other quiet method. See that the tongue is well forward.

17. Do not cool the patient by exposure and wet surroundings.

18. Being first assured that he can swallow a teaspoonful of water, feed him, if you like with stimulus, during the expiration, but not the inspiration.

19. Give to all painful surgery, without exception, the benefit of anasthesia; but a patient equivocally exhausted by long disease—of the bladder, or of a joint, for example—or an habitual inebriate, may require care; without which, protracted narcotism may gradually depress his pulse beyond the rallying point. On the other hand, a healthy laborer, who reaches the hospital some hours after a railroad accident, cold and literally pulseless at the wrist, from hemorrhage and exposure, is, as a rule, stimulated by the ether during and after at least one amputation.

20. Notwithstanding every expedient, there is occasionally an untoward subject who is habitually tetanic and livid, whenever etherized; or, more rarely, one whose respiration is notably intermittent before he becomes insensible. The latter requires attention. In children, it may added, anæsthesia is cumulative.

Such are some of the minor considerations and prompt precautions which collectively determine the question of life or death in the exceptional emergencies of anæsthesia by ether. Many of them apply with equal force to chloroform; but against the shock of chloroform and its sequences, whether "chloroformic syncope," "cerebral anæcia," or "cerebral congestion," precaution avails nothing.—*Nashville Journal of Medicine and Surgery.*

---

